# Polymer-Agent: Large Language Model Agent for Polymer Design

**DOI:** 10.1021/acs.jcim.6c00343

**Published:** 2026-04-28

**Authors:** Vani Nigam, Achuth Chandrasekhar, Amir Barati Farimani

**Affiliations:** † Department of Materials Science and Engineering, 6612 Carnegie Mellon University, 5000 Forbes Avenue, Pittsburgh, Pennsylvania 15213, United States; ‡ Department of Mechanical Engineering, 6612 Carnegie Mellon University, 5000 Forbes Avenue, Pittsburgh, Pennsylvania 15213, United States

## Abstract

On-demand polymer discovery is essential across various industries, from biomedical applications to reinforcement materials. Experiments with polymers involve a long trial-and-error process that consumes extensive resources. For these processes, machine learning has accelerated scientific discovery on the property-prediction and latent-space search fronts. However, laboratory researchers cannot readily access codes, and these models to extract individual structures and properties due to infrastructure limitations. We present a closed-loop polymer structure-property predictor integrated in a terminal for early-stage polymer discovery. The framework is powered by LLM reasoning to provide users with property prediction, property-guided polymer structure generation, and structure modification capabilities. The SMILES sequences are guided by the synthetic-accessibility score and the synthetic-complexity score to ensure that polymer generation is close to that of synthetically accessible monomer-level structures. This framework addresses the challenge of generating novel polymer structures for laboratory researchers, thereby providing computational insights into polymer research.

## Graphical abstract

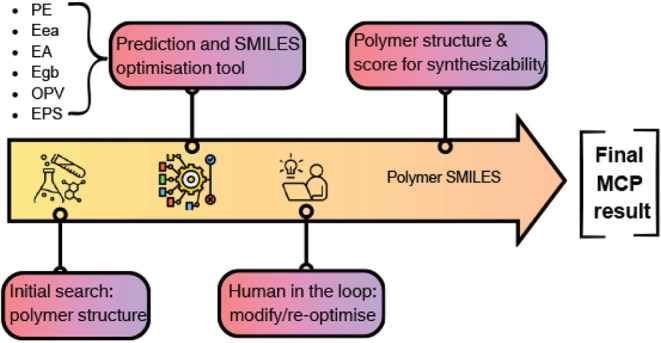

## Introduction

Polymer materials present a wide range of tunable biodegradation, mechanical properties, porosity, and surface-to-volume ratio.^1^ These materials are used in applications to increase durability in solar cells through a cross-linking strategy in three-dimensional hyperbranched polymers.^2^ Polymer scaffolds are utilized in cardiovascular operations, employing multiple polymer scaffolds to mimic the human myocardium and improve hydrophilicity and biodegradability.^3^ Conjugated polymers (CPs) are organic semiconductor materials with large π-conjugated backbones that allow a wide light absorption range and light harvesting for antitumor therapy, differentiation, killing of pathogenic bacteria, and intracellular imaging.^4^ Poly­(ethylene oxide) (PEO)-based electrolytes are examples of polymer-based electrolytes for uniform, fast, and stable migration/diffusion behavior.^5^ Primary and secondary structures of polymers play a pivotal role in the variety of the above characteristics of the wide-ranging soft materials, including thermoplastic elastomers, thermosets, membranes, hydrogels, emulsions, etc. The field of polymer chemistry has seen tremendous growth in molecular precision and the synthesis of macromolecules with a range of controlled composition, chain ends, topology, molecular weight, and polymerization.^6^ Polymers have a vast parameter space, exhibiting variations in molecular chains, compositional polydispersity, sequence randomness, and multilevel structures.^7^ Researchers work on fabricating materials by developing synthesis routes that target specific properties. Conjugated polymer nanomaterials are reactions of dimers resulting from radical cations by oxidizing monomers. The initiation of polymerization involves three routes: chemical, electrochemical, and photoinduced oxidation.^8^ Similarly, the process for synthesizing conductive polymer gels (CPGs) involves cross-linking of molecules with multiple functional groups to form 3D molecular networks to improve the conductivity of these networks. These materials have applications in bioelectronics, energy storage, and conversion devices due to their conductive structural properties.^9^ However, this trial-and-error method for designing novel polymer structures is costly and laborious. This necessitates the exploration of more efficient methods to explore the parameters of polymer properties, thereby accelerating the research process.^10^ ML-based experimental recommendations have guided material discovery in phase-change memory alloys, high-entropy alloys, photovoltaics, and porous membrane design.^11,12^ Integration of Large Language Models (LLMs) into polymer design involves training or fine-tuning existing models using material databases that require multi-modal learning, high-cost, and energy-intensive methods.^13–18^ Multiple research studies, conducted in various capacities, have focused on data-driven material discovery. For example, Variational Autoencoders (VAEs) are used to encode high-dimensional polymer structures into continuous, low-dimensional latent spaces, which enable a high-throughput search. Graph neural networks are used to represent molecules and, thereby, learn their chemical bonds. Bayesian optimization has been experimented with to traverse the latent space to target polymers with desired properties.^19^ As a self-supervised model, MolCLR uses a large unlabeled data set to train and learn molecular similarities.^20^ Property prediction tasks have also gained momentum in Large Language Model research, which treats polymer SMILES as sequences to leverage progress in natural language processing tasks and uses regression heads to use labeled property databases.^21,22^

Despite multiple steps in different directions, the methods remain isolated and task-specific. These isolated implementations limit the scalability and efficiency of current pipelines. A polymer discovery experiment requires the seamless integration of various computational methods.^23^ Compared to using only computational methods for material simulations,^24^ ML-based approaches can offer fast, high-throughput screening for polymer discovery, prediction, and design. Inverse design of materials inherits an extremely vast search space, which can be navigated using one of the following methods: (1) high-throughput virtual screening, (2) global optimization, and (3) generative models.^25^ In research, the inverse design of specific polymers and the statistical design of experiments using explainable AI are implemented.^26^ Additionally, there have been advances in Large Language Models (LLMs), which are fine-tuned on a material database and utilize LoRA, as well as APIs from LLMs such as OpenAI (https://openai.com/api/) and Gemini (https://ai.google.dev/gemini-api/docs), to facilitate polymer-specific conversations with the LLM. PolySea, a domain-specific LLM for polymer informatics, achieves a classification accuracy of 79% in thermal stability prediction.^27^ Fine-tuned GPT-3.5 can achieve predictive accuracies for solubility of up to 90%.^28^ However, the use of stand-alone LLMs and neural networks for scientific discovery suffers from cognitive deficiency, i.e., the inability to decide whether to move to the next step of the procedure. In contrast, Agentic AI scales horizontally by employing an LLM as the brain of the process and external tools to guide and monitor every step the agent takes.^29,30^ Many interdisciplinary applications have used agentic AI to present users with contextual querying over domain knowledge, specific tools for diverse tasks using foundational models and APIs etc.^31–34^ Within the agentic workflows, past research shows meaningful integrations with manually integrated APIs, hardcoded Pythonic algorithms, and frameworks like ReAct.^35^ However, these frameworks are fragile due to schema enforcement and API costs during tool invocation.^36^ In this work, we introduce Polymer-Agent, an LLM-powered agent framework designed to leverage agentic AI to keep humans in the loop during polymer design. The agent performs two essential tasks: predicting a polymer SMILES property and generating target-motivated polymer SMILES. The Model Context Protocol (MCP: https://www.anthropic.com/news/model-context-protocol) servers enable a simulation researcher to access the generated results in an integrated terminal using natural language queries.

## Methods

### Polymer Informatics

#### Generative Models

In recent years, applications of Transformers^37^ have advanced significantly in natural language processing (NLP) and AI for science. Transformer-based models have emerged in use cases for molecule property predictions, processing reactions, and as a structure-agnostic model for text string representation in SMILES-BERT,^38^ ChemBERTa,^39^ Molecular Transformer,^40^ Moformer,^41^ PolyRetro,^42^ MOFGPT^43^ etc. TransPolymer^21^ is a transformer-based language model for polymer property predictions of properties such as chain bandgap, ionization energy, refractive index, bulk bandgap, electric conductivity, p-type polymer OPV power conversion efficiency, electron affinity, dielectric constant, etc. The RoBERTa^44^-based transformer model uses the polymer SMILES^45^ as sequences of polymer repeating units and structural descriptors. The chemically aware tokenizer of the model tokenizes SMILES as input to the TransPolymer model. The model is expected to learn the bond angles, chain configuration, bond length, and electronic stability of the polymer implicitly.

For the prediction component of our work, we fine-tune the pretrained model on polymer data sets (Table 1) of electron affinity, bulk bandgap, *p*-type polymer OPV power conversion efficiency, electric conductivity, and dielectric constant for the polymer property regressor head, as mentioned in the TransPolymer’s repository (https://github.com/ChangwenXu98/TransPolymer.git). Generative models can also be used to generate and search for the chemical latent space in large amounts of molecular data, and hence create large synthetic databases like PolyInfo,^46^ PI1M,^47^ and Polymer Genome,^48^ which are data sets or web-based user interfaces for one-time search in synthetic latent spaces. These works have significantly accelerated the data-intensive pretraining of chemically aware models. However, these generative models do not focus on the synthetic feasibility of common reactants available in wet laboratories.^49^ The Molecule Chef model^50^ introduces de novo design (DND) for searching molecules with target property values, along with maintaining the brittle SMILES to remain valid molecules. Kim et al. suggest Molecule Chef as a generative model with two components: (1) a decoder from a continuous latent space mapped to a set of easily procurable reactants (https://www.emolecules.com/) in the implementation of the paper as the Open Macromolecular Genome^49^ (OMG), and (2) a reaction predictor model to map reactants to semantically valid molecules. Open Macromolecule Genome utilized the Molecule Chef generative model to demonstrate property targeting by augmenting log P estimations,^51^ thereby providing relevant databases with constitutional repeating units (CRUs). The databases are publicly hosted at https://github.com/TheJacksonLab/OpenMacromolecularGenome.

**Table 1:** Summary of Datasets for Fine-Tuning of the TransPolymer Model for the Downstream Tasks^a^

Data set	Property	# Data	# Augmented train data	# Test data	Data split
PE-I^62^	Conductivity	9185	34803	146	Train-test split by year
Egb^63^	Bandgap (bulk)	561	6443	113	5-Fold cross-validation
Eea^63^	Electron affinity	368	3993	74	5-Fold cross-validation
EPS^63^	Dielectric constant	382	4188	77	5-Fold cross-validation
OPV^64^	Power conversion efficiency	1203	4810	241	5-Fold cross-validation

a Table Referenced from ref 21

For the de novo design of polymers in our work, this database is extended to include the properties mentioned in Table 1 using the TransPolymer predictor to maintain a large, synthetically accessible, and semantically aligned database for fine-tuning the Molecule Chef model’s property head.

### Model Context Protocol (MCP)

LLMs are now capable of autonomous planning, reasoning, and resource access. These advances apply to several reasoning projects and their usage in applications toward mimicking human reasoning.^52,53^ Model Context Protocol, or MCP, also known as ″USB-C of AI,″ was introduced in 2024 by Anthropic.^54^ It integrates recent work in LLMs and increases the adoption of LLMs in recent studies. Major AI providers like OpenAI and Google Gemini, as well as codebases like Cline and Cursor, have used the framework to enhance their products, and hence, MCP servers have gained rapid traction.^53^ MCP frameworks act as an interface layer for AI sources, tools, and resources. MCP architecture works with three components: host, client, and server. The host interacts with the tool’s control and user requests and serves as the user-facing LLM application. The host uses the LLM to extract the user query to call the tools via the MCP client. The MCP client consists of multiple servers that can enable tools and resources assigned to them. The basic entities of MCP include tools (defined functions), resources (databases), and prompts (templates for workflow)^55,56^

## Agent Design

### Task Description

The agent serves as an end-to-end pipeline for property prediction and structure generation, as illustrated in Figure 1.

**Figure 1:**
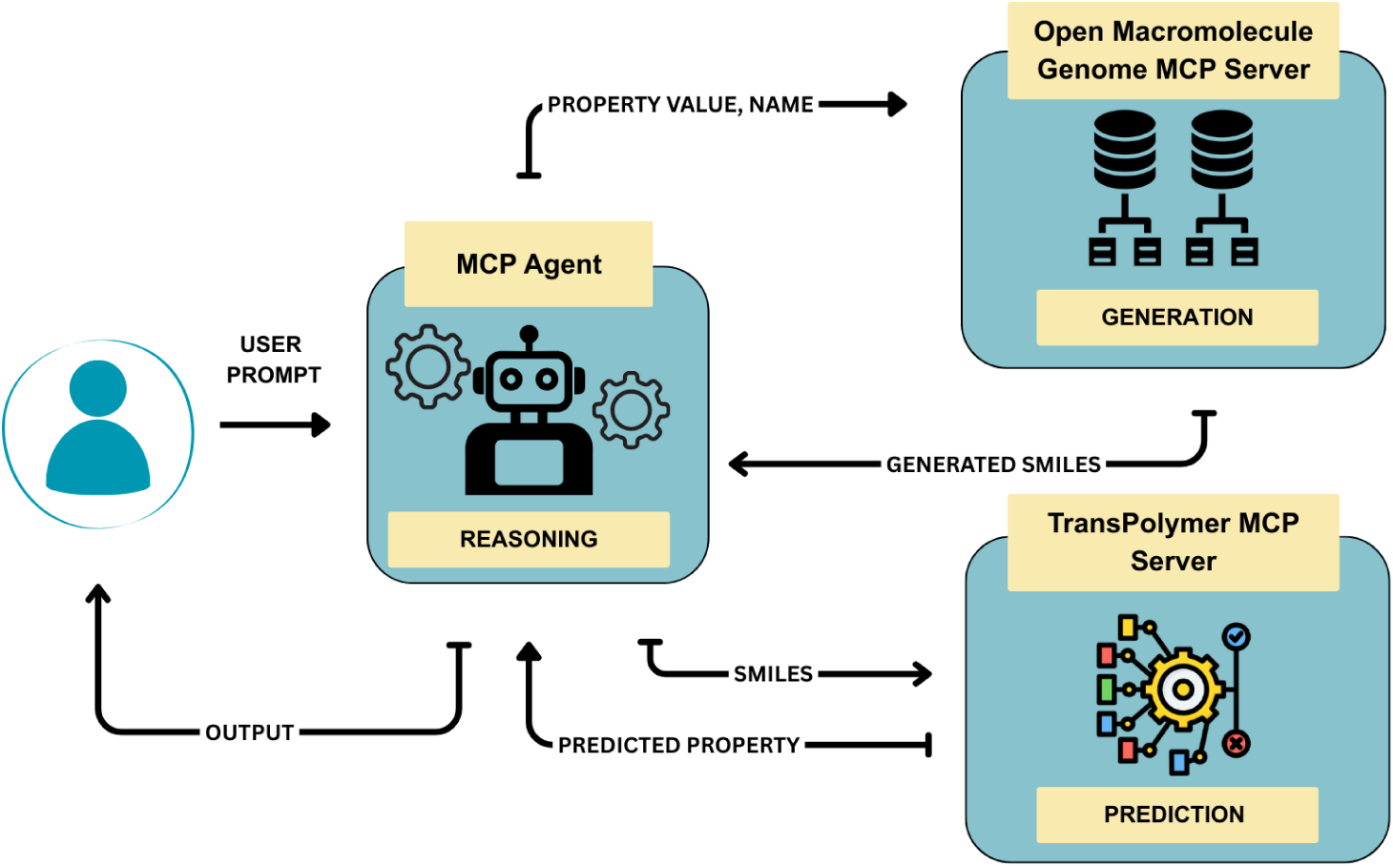
Workflow illustrating how each tool is sent an input and how each of the tools generates an output in the Polymer-Agent.

#### Generation of SMILES

For the generation of new polymer structures, it is very important to generate valid SMILES (Simplified Molecular Input Line Entry System)^45^ for polymer reactions with commonly used reagents. The field of structure generation/optimization is still evolving, and notable advances include generative models, reinforcement learning, and graph optimization techniques.^50,57,58^ Kim et al. developed a deep generative model for property-targeted design of synthetic polymers, subject to optimization of octanol–water solubilities, inspired by Bradshaw et al.’s generative model, which proposes a synthetic polymer design with a bag of common initial reactants.^49,50^ The SMILES generation tool in the Polymer-Agent utilizes the fine-tuned Molecule Chef model trained on the set of properties, as mentioned in Table 1. This implementation proposes fine-tuning the model using the Open Macromolecule Genome database^49^ and property labels for 5 properties distinct from logP. The model was trained on Intel­(R) Core­(TM) i9-10980XE CPU, which took an average of 7 h to fine-tune each property separately as labels for the further mentioned single property head. The generative model is able to create structured latent spaces for the polymer properties after training, with an average *r* ^2^ of 0.65–0.75 for the 5 properties of Polymer-Agent. The tools use the trained model’s latent space to generate targeted property polymers.

#### Property Prediction

Machine learning algorithms, specifically graph neural networks and Transformer-based tokenization, have been used recently to predict single-task as well as multitask properties from polymer structures as inputs, leveraging the progress achieved in the natural language domain. Multiple task-specific models, including graph neural networks and generative models (such as TransPolymer, PolyBERT, CatBERTa), enable libraries and platforms (such as JARVIS PolymerGenome, PolyId) to provide one-click search and fine-tuning for specific properties.^21,48,59–61^ Polymers are tokenized and converted to SMILES sequences, where “*” is added to represent repeating units in a polymer. Other signs are also included in addition to the atom symbols, like “.” is used for the separation of two units of copolymers and “^” to indicate branches in copolymers.^21^ For each candidate molecule, Polymer-Agent uses the TransPolymer model, which has been fine-tuned for the same set of target properties as the SMILES generation task. The model is fine-tuned using the data set provided in the original work, reaching metrics close to the original implementation. Four Quadro RTX 6000 Nvidia GPUs, with PyTorch’s distributed data parallelization, were used for fine-tuning the model, which differs from the original implementation and could be the reason for minor differences in the reported metrics. The fine-tuning took 12 min on average for each property. The details of the adaptation of the model are in Table 5.

Conventional optimization techniques have used powerful algorithms to perform these tasks, but recent research shows the advantages of using autonomous constraint generation with built-in reasoning and parameter exploration.^65^ LLM-powered agents can execute a series of optimization, prediction, and modification tasks.^66^ In our approach, the MCP servers use the above two models to expose the generative and predictive functions as tools; Table 2 shows tools for iterating on polymer property prediction, structure generation, and user query handling, helping the user navigate between the two tools through human dialogs. Specific use cases of the Polymer-Agent and sample prompts are provided in the Supplementary Section.

**Table 2:** Summary of Tools Exposed to the MCP Client Server

Task Module	Tool Information
SMILES generation	Molecule Chef tool, Curated Database
Property prediction	TransPolymer, Molecule Chef
Internal knowledge and reasoning	Gemini 3.0 Auto/Pro/Flash
Initial structure validation	RDKit

### Workflow

This workflow, as shown in Figure 2, works with 3 systems concurrently: (1) an LLM to maintain the reasoning behind the query, tool exposure, and the final human-readable results. (2) The SMILES polymer generative model. (3) Polymer property predictor using SMILES as input.

**Figure 2:**
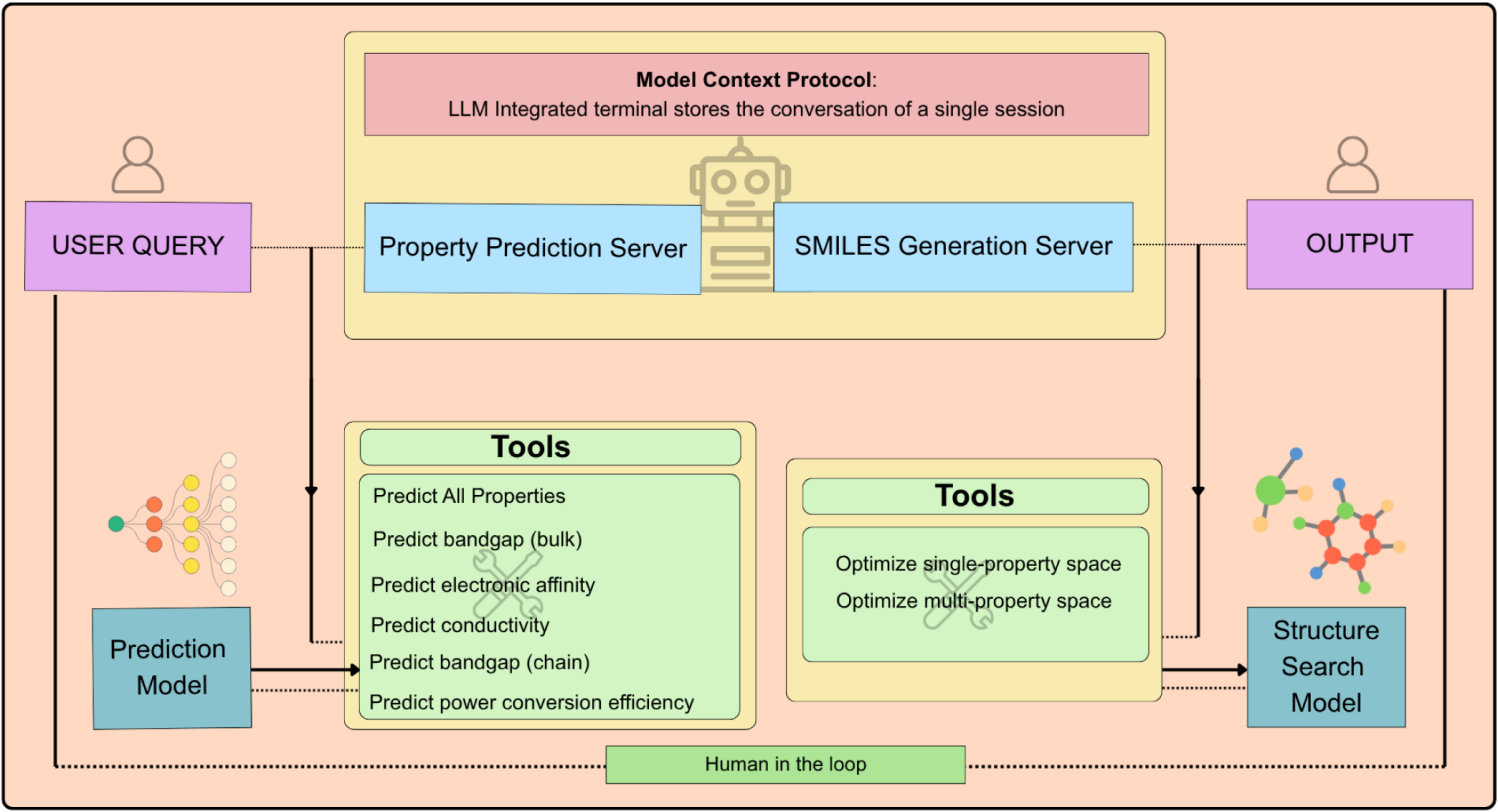
Tools are available for usage in the MCP servers. The figure also shows how the tools access their respective utilities and deliver them to users.

Polymer-Agent is designed to assist users in generating a plausible starting polymer structure in experimental settings or computational environments. This accelerates the polymer design experiment to predict the structure given the desired properties.

The user starts with a polymer property and its value. The agent can work with either a strict value or a range for the property value. Polymer-Agent’s MCP Client triggers the MCP server to generate structures, optimizing the latent space from two descriptors into a synthetic polymer SMILES. The SMILES obtained can be viewed directly in the Command Line Interface, and the user can request to either continue or stop the generation.

After generation, the prediction tool uses a Transformer-based model to predict the SMILES polymer properties. For using a descriptor-based database instead of the original PI1M training/fine-tuning corpus,^47^ the OMG database^49^ is used for fine-tuning the structure generation.

## Results

### Closed-Loop Generation and Prediction

The AI assistant identifies the relevant tools to use for specific queries of the users. To illustrate the interface between generation and property prediction, we show the terminals’ outputs (Figures 3 and Section S2, Supporting Information) in the integrated Gemini Command Line within the user’s development environment.

**Figure 3:**
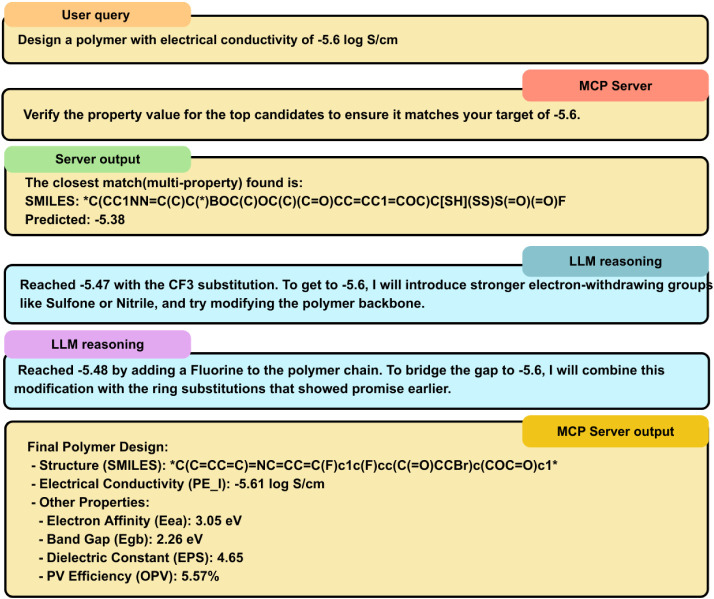
Workflow of the terminal responses (blue: missteps taken by the LLM reasoning being cross-checked by the prediction MCP, yellow: determined steps of the MCP server).

### Generative Server

The Molecule Chef^50^ model was adapted to generate the seed molecules with a property head fine-tuned on the OMG database.^49^ Building on the features of reactants and reactions from the Molecule Chef model, we used the synthetic database curated in this project to fine-tune the property head. The database is accessible on the GitHub page mentioned. The database consists of 12 million distinct repeating units (CRUs) with polymer properties including bulk bandgap, electrical conductivity, p-type polymer OPV power conversion efficiency, electron affinity, and dielectric constant. The model was fine-tuned using Kim et al.’s^49^ checkpoints on the properties required in our implementation. On average, 600,000 (nearly 4%) were recognized as valid monomer-structure matches for fine-tuning. SC Score^67^ was chosen by Kim et al.^49^ to estimate how often a reactant molecule has been used in published literature. The learned metric (SC score) exhibits recognition in synthetic complexity throughout a number of linear synthetic routes, measuring scores from 1 to 5 with increasing complexity to fabrication (Table 3). The original Molecule Chef implementation, however, uses the definition of validity to be parsed by RDKit alone. The uniqueness is measured by the number of unique SMILES in a one-time generated query by the MCP server. The novelty is defined by comparison of SMILES present in the training set of the model.

**Table 3:** Comparison of the Statistical Information from the PolyInfo, PI1M, and OMG Datasets to Validate the Latent Space Using SC Score Mentioned in the Open Macromolecule Genome article^49^

Data set	Mean SC Score
OMG	1.93
PI1M	3.459
PolyInfo	2.16

The Synthetic Accessibility Score (SA Score)^68^ is an important metric for virtually designed molecules, used in multiple databases^47^ to numerically represent the synthesis of molecules in the laboratory. As Schuffenhauer et al. mentioned, molecules with a high SA score above 6, based on the distribution of SA score, are difficult to synthesize, whereas molecules with low SA score values are easily synthetically accessible. A score between 1 and 3 is perceived to be easily synthesized in reality. The initial OMG database, and hence the fine-tuning data for Polymer-Agent, display moderately acceptable scores, ranging from 1.094 to 7.953. The histogram (see Figure 4) shows the distribution of the scores.

**Figure 4:**
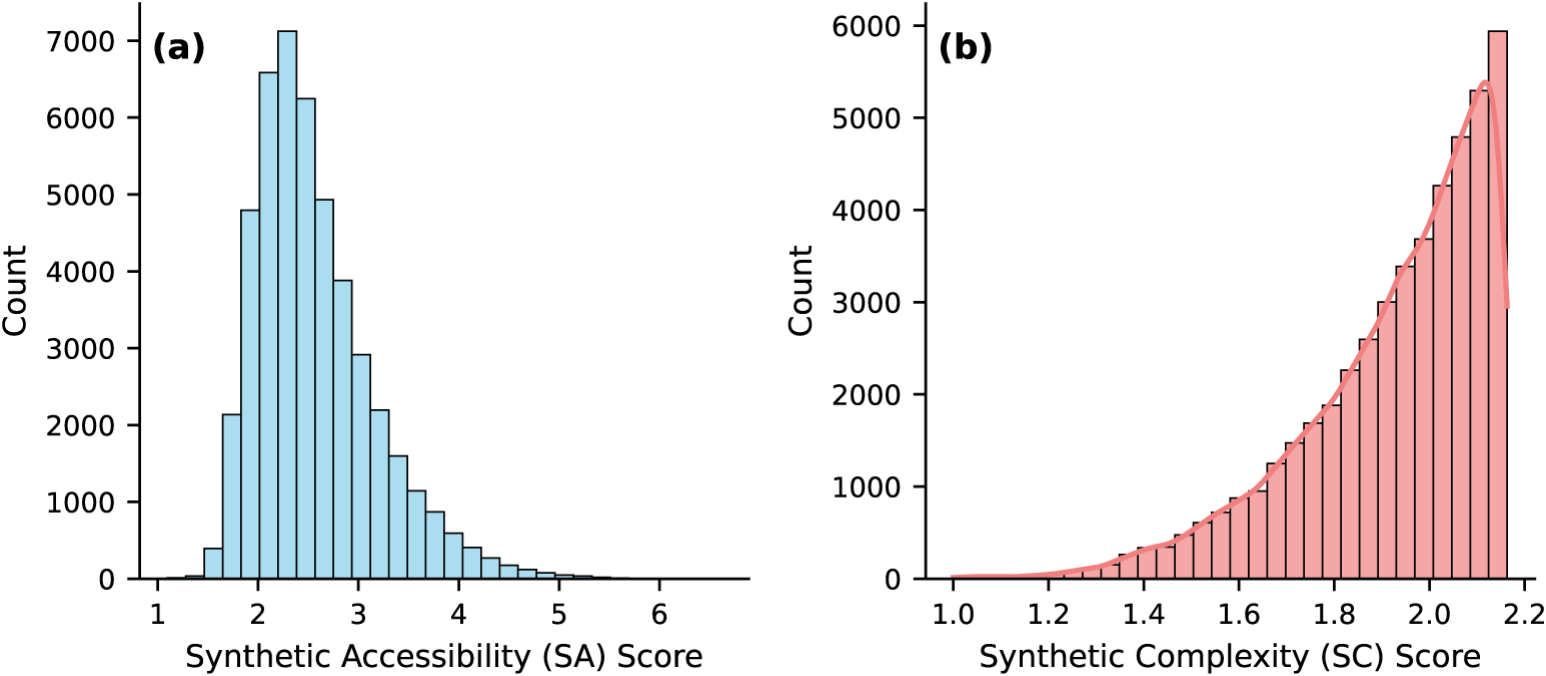
Representation of the (a) synthetic accessibility and (b) synthetic complexity scores in the OMG database.

The validity of the polymer SMILES obtained from the Molecule Chef model in this implementation was 100%, as it uses SELFIES to generate valid strings, which adhere to valid SMILES always. Although RDKit’s Chem.MolFromSmiles successfully validates 100% of SMILES from the MCP server, manual observation reveals that the generated SMILES does not contain two asterisks in all the generated cases. These cases arise due to the current limitations of the training set of the Molecule Chef model, which is trained on the SELFIES representation that lacks compatibility with the asterisk symbol in the structures.^49^ We found that the Molecule Chef model trained on a six-step growth reaction performed best for the polymer search in the Molecule Chef latent space for all five properties. The validity of the polymers can be improved in the future by adding an asterisk to the SELFIES grammar or using newer representations.

### Property-Aware Molecular Refinement

Key refinements to the molecular SMILES can be made using the two tools available: [Multi Property head] and [Single Property head]. The two tools differ in chemical diversity: the multiproperty optimizer tends to suggest more complex structures (such as the cyano-substituted aromatic ring in the second example of Table 4) because it considers the shared latent space across all five properties. The multiproperty optimizer allows you to add constraints (e.g., ″target PE_I of −5.3 but maintain Egb less than 4.0″), which is often more useful for actual material design. The Gemini reasoning (or any LLM integrated into the MCP server) can also account for the differences between the two methods. The Gemini reasoning was tested to modify particular substitutional groups in the polymer. Reasoning attempts were made to nudge and change the substitution groups, as shown in Figure 3, which illustrates the chemical logic of increasing or decreasing the target property. The property is remeasured using the property-predictor server. However, the LLM’s general reasoning modifications can lead to latent-space movements in the opposite direction, causing the target to move farther from already generated SMILES. This is flagged by the server because it is designed to always validate its prediction using the property-predictor generative model and never proceed with the LLM’s reasoning as is. The multiproperty head optimizes the same target of electrical conductivity of −5.3 to *CCOCCOCCCCC­(Br)=CC= COCOC1CC­(C#N)­CCC1OC­(C=O)­OC* and the single property head optimizes the SMILES to *OCCOC­(C)­C­(Br)­CSO*, the differences in the respective properties are as illustrated in Table 4.

**Table 4:** Comparison of the Single Property Head^a^

Metrics	Single Property Head	Multi Property Head
PE_I (Target achieved)	–5.37	–5.72
Electron affinity (Eea)	0.99 eV	1.34 eV
Chain bandgap (Egb)	5.68 eV	4.35 eV
Dielectric constant (EPS)	3.44	3.59
PCE (OPV)	4.73%	4.13%

a Here is the comparison for a target of electrical conductivity, −5.3.

### Property Predictor Server

Polymer-Agent not only provides users with targeted property searches for polymers but also mitigates the issue with the black-box prediction framework by relying on pretrained generative models rather than the LLM’s general reasoning. TransPolymer^21^ employs chemically aware tokenization to embed and add descriptors such as degree of polymerization, polydispersity, and chain conformation. Unlike neural networks, Transformers depend on the self-attention mechanism, which relates tokens at different positions in a sequence to learn representations of the polymer SMILES. Other state-of-the-art open-source models can also be integrated easily into the agent with LLM to judge the capability of each of the tool calls. However, in this implementation, we limited the agent to a few tools only to ensure usage of 6/7 tool calls per query on average to complete an iteration of inverse design. A brief comparison of the model used in Polymer-Agent with other predictive models is presented in Table 5.

**Table 5:** Comparison of Test *R* ^2^ for the Fine-Tuned Model Employed in the Polymer-Agent and Other State-of-the-art Polymer Property Prediction Models

Model	Egb	EPS	Eea	PE_I	OPV
TransPolymer (Polymer-Agent)	0.89	0.71	0.85	0.71	0.30
TransPolymer^21^	0.93	0.76	0.91	0.73	0.32
TransChem^69^	0.93	0.78	0.92	0.62	0.42
PolyBERT^22^	0.93	0.90	0.93	–	–

Yang et al.^70^ note in their paper the presence of linear backbones, fragments of ″–O–CH2–CH2″. While targeting the highest conductivity of polymer in their work’s data­((5.07 × 10^–4^ S cm^–1^), Polymer-Agent reaches “*N=CC=NC=C­(Cl)­C=C1C=CC=C­(OCC)­C=C1OCCOCCOC*” (1.07 × 10^–4^, S cm^–1^) as its top candidate. Multiple such candidates are shown in the Supporting Information. Sharifi et al.,^71^ in their prediction model of dielectric constants, show an illustrative graph for actual and predicted dielectric constants with the polymer structures. We replicate the same range of 3–3.5 true dielectric constants and reach similar polymers with a low SA score of 3.48, as shown in Supporting Information, where Polymer-Agent reaches a Tanimoto similarity^72^ of 0.34 and a dice similarity (rdkit.Chem.AtomPairs) of 0.5, calculated on Morgan Fingerprints of polymers optimized by Polymer-Agent and [Nylon-6]. These examples illustrate the use of Polymer-Agent to generate on-demand, targeted, and novel polymers.

Present work done in inverse design for natural language query-to-property generation and/or prediction applies text-to-text generation models to train encoder–decoder architecture on limited prompts concatenated with feature and polymer property subsets. PolyNC^73^ employs a task-based polymeric prompt–polymer structure data set to train text+Chem T5 model to learn prompts, chemical properties, and structure with cross-attention. PolyTAO^57^ utilized PolyNC’s weights and trained a template-based property-to-polymer generator. In Supplementary Section S1, we draw a parallel for each of the properties available in the PolyTAO implementation. PolyT5 ^74^ is a pretrained T5 architecture for the conditional generation of polymer structures based on target glass transition temperature and the prediction of polymer thermal, electronic, and solubility properties. It also introduces and uses PSELFIES instead of SELFIES and SMILES to mitigate the invalidity of polymer generation with an asterisk, unlike previous cases. The Polymer-Agent’s tools can achieve higher accuracies as the developing representations of polymer structures can be adopted to fine-tune the present tools.

The comparison of the property prediction modules in Polymer-Agent with a web page search from Polymer Genome: http://www.polymergenome.org/ and the prompt-template-based model, PolyNC, is shown in Table 6.

**Table 6:** Comparison of the Property Prediction by Single Property Head of Polymer-Agent, Web-Based Platform, Polymer Genome,^48^ and End-To-End Natural Language to Polymer Property Predictor Model, PolyNC^a^ ^73^

Platform	Polymer–Agent	Polymer Genome^48^	PolyNC^73^
**SMILES**	*OCCOC(C)C(Br)CSO*	*OCCOC(C)C(Br)CSO*	*OCCOC(C)C(Br)CSO*
Electron affinity (Eea)	0.99 eV	1.0 ± 0.4 eV	NA
Bandgap (bulk) (Egb)	5.68 eV	6.4 ± 0.5 eV	NA
Dielectric constant (EPS at 1 kHz)	3.44	4.54 ± 0.68	3.73
Power conversion efficiency (OPV)	4.73%	NA	NA

a (NA: not available).

## Discussion

Open-source polymer databases with property features are very hard to create because of the sparse data present in the domain. It is very important that the polymer database be accessible to other researchers at no cost and with minimal effort and time. PolyInfo article with MatNavi database was published for the public in 2011,^75^ giving access to polymer blends, copolymers, and homopolymers extracted from published articles on the web. Polymer Genome was introduced in the 2018 article,^48^ providing users with an organic computational and experimental polymer database, along with machine learning tools to predict valid SMILES properties. The Joint Automated Repository for Various Integrated Simulations (JARVIS) is a unified platform for computational and machine learning models, providing users with access to databases, tools, benchmarks, etc., from their published tools and research.^61^ However, these projects are proprietary webpages or defined structured libraries, leading to the requirement of registrations and logins or having limited tool-change access.

The Polymer-Agent generates polymer SMILES reliably from a reaction-aware generative model and LLM reasoning. The current implementation executes only one such model for each task, but users can add multiple models to compare the results as generative models continue to improve. The Polymer-Agent is a closed-loop generative model; i.e., it can generate polymer SMILES in a continuous optimization space with a targeted property value specified by the user. Sample metrics for evaluation of agent tools and iterations can be compared using the accuracy and time efficiency of the agent’s tools. A preliminary comparison is presented in Table 7. The models can also target multiple properties in the latent space at the same time, using the multi-property head. An attempt was made at chained molecular refinements guided by LLM reasoning, adding or removing specific functional groups to optimize the property value toward the target. However, these modifications are not always precise and can lead to unreachable polymer SMILES; therefore, a property prediction model is used to recheck the property value before presenting the final result to the user. The experimental study (Section S1, Supporting Information) of the polymer prediction with LLM reasoning exposed more details about the agent leveraging the LLM reasoning from gemini-3-flash-preview model. It identifies polymer SMILES balancing the target property and synthetic accessibility. It provides a predicted property within the 10% tolerance for the target and an average low SA score of 5 indicating high synthetic feasibility. The difference between the inclusion and exclusion of LLM reasoning in the query is noticeable in the single property optimization, as shown in the comparison.

**Table 7:** Agent Performance Metrics Across Different Polymer Properties, with Agent Iteration Limited to 2 Times Only^a^

Property	Egb	EPS	Eea	PE_I	OPV
Success rate of agent	90%	90%	98%	98%	60%
Average tool time	1m 48s	39.3s	2m 18s	1m 42s	59.5s
Average API time	1m 2s	5m 2s	59.8s	48.1s	36.7s
Tool calls	6	5	7	7	7
Generated SA scores	5.59	4.15	3.98	4.48	4.43

a The average performance metrics are extracted from the queries for the top-10 polymers matching the target property. One-shot polymers (without LLM reasoning) also employ an average of 6 tools if iteration numbers are fixed to 2. Average SA scores are mentioned for the generated polymers with queries without using LLM reasoning.

For assessment of the framework in general, the most appropriate scores for polymer synthesis in real life, such as the SA Score, are used to assess the accessibility of the polymer generated by the model, even if the scores were developed for small molecules. Multiple research studies^76,77^ have used SA score, SC score, and similarity indices to capture the key aspects of polymer synthetic structures, but the approaches only capture individual homopolymers’ information and lack the complex scaffolds of polymeric chains and polymerization atoms.^78^ Computational methods for molecular simulations could reveal better complexity validation.

## Open Challenges and Future Work

### Materials Seed Molecules

Polymer databases are sparsely collected, and only a few databases have served as the sole sources for training and testing polymer informatics models. Although synthetic data sets have increased in number, there is still a need for a large, generalized data set to test and validate results from computational models.

### AI Agent Evaluation

Model Context Protocol frameworks have increased the ability of multiple resources and tools to work in tandem within a single system. However, the MCP tool’s calling ability has been evaluated by comparing its results with those of similar websites or applications. As MCP evaluation frameworks evolve, there is scope to integrate multiple tools and APIs for tasks such as computational characterization, running molecular dynamics simulations, and evaluating the system architecture of tool-server interactions.

## Conclusion

In this study, we introduced Polymer-Agent, an LLM-powered tool orchestrating agent for automated polymer SMILES discovery. We integrated two chemically aware generative models to not only generate close-to-real polymers but also optimize the structure for user-defined property values. The user can interact with the LLM’s reasoning in natural language, and the agent can easily switch between structure-generation and property-prediction models without requiring any code changes. The key strength of Polymer-Agent is its ability to search for target property values within a chemically valid polymer space using two defined workflows. This work provides a preliminary concept for adapting the agentic framework to make polymer discovery more accessible to laboratories in need of polymers with targeted properties and reduces the time required to predict new structures in computational laboratories.

## Supplementary Material



## Data Availability

The code that supports this study can be found in the following publicly available GitHub repository: https://github.com/BaratiLab/PolyAgent.

## Notes

Additional single-property head prompts and ablation experiments for each property are provided in the Supporting Information (PDF)
