# Supplementary material for: Polymer-Agent: Large Language Model Agent for Polymer Design
Source: J Chem Inf Model. 2026 Apr 28;66(9):5145–54. doi: 10.1021/acs.jcim.6c00343 (PMC13169346; doi:10.1021/acs.jcim.6c00343)
Supplement: Supplementary file 1 [file ci6c00343_si_001.pdf]

# Supplementary Information

## Polymer-Agent: Large Language Model Agent for Polymer Design

Vani Nigam,† Achuth Chandrasekhar,‡ and Amir Barati Farimani*,‡

†*Department of Materials Science and Engineering, Carnegie Mellon University, 5000 Forbes Avenue, Pittsburgh, PA 15213, USA*

‡*Department of Mechanical Engineering, Carnegie Mellon University, 5000 Forbes Avenue, Pittsburgh, PA 15213, USA*

E-mail: barati@cmu.edu

Section S1 provides comparatives for target property generations (top–10 and top–1) from the MCP server along with an ablation study with LLM reasoning. Section S2 provides exact prompt–results from the Polymer-agent.

# S1. Top-10 and One shot target property search

The results section mentioned the uniqueness and novelty of the generated SMILES from the MCP server for every query with the following constraints. These examples also describe the application of general LLM to modify the functional groups alternatively. The results for each property are as described in this ablation study. All the queries were generated from the simple prompt of ”generate [top–10] / [a valid] SMILES with [property name] of [property value] with [value] tolerance and low SA score, use generation and prediction MCP, and try only twice for iteration”, to maintain uniformity.

**Bandgap (bulk, Egb)** – For one–shot polymer search, a polymer within the 10% tolerance was generated in the second iteration, which was orchestrated by the agentic tools by changing the number of seeds and steps for the Molecule Chef model. With LLM reasoning inclusion, agent was successful in balancing the target bandgap and synthetic accessibility. It provided a predicted bandgap within the 10% tolerance for the 6.0 eV target (5.4 - 6.6 eV), and a low SA score of 3.16 (average), indicating high synthetic feasibility. Additionally the gemini– 3 –flash provided polymer functional groups which are recognized in LLM's web search. For top – 10 polymer generation, we noticed a decline in the validity of the SMILES as it generated only 9 unique SMILES, out of which, although all SMILES could be parsed with RDKit, only 6 had two [*]. PolyTAO,[1] a recent inverse design implementation, works in a similar range of 13 out of 15 novel generations over 3 iterations.

**Electron Affinity (Eea)** – Top–1 polymer for finding electron affinity for n –type conjugated polymer was identified in the second iteration using the single property optimization tool, which proved more effective in converging on the target property value generating valid polymer with SA score of 3.98 and Eea of 2.8 eV (within 10% of 3.0 eV). For one shot polymer search with LLM reasoning, the agent successfully reached the conjugated ketone system which provided it with necessary electron affinity target between 2.8 and 3.0 eV and low SA score of 3.29. For top –10 polymers, the MCP could generate 10 unique polymers with 10% tolerance to target value, out of which, although all SMILES could be parsed with RDKit, only 6 had two [*]. PolyTAO[1] works in a similar range of 14 out of 15 novel generations over 3 iterations.

**Conductivity (PE_I)** – For top polymer search, the agent struggled to reach perfectly insulating polymers but was able to reach a polymer in the conductivity target range of –5.0 to –6.0 log S/cm with SA Score of 4.48 (Relatively low complexity). This candidate was generated in the first iteration and confirmed using the TransPolymer predictor. Based

on the target conductivity of –5.0 log S/cm and a 10% tolerance (range between –4.5 to –5.5) using LLM reasoning. It reasons and adds the aromatic amide linkages to provide the necessary electronic decoupling to reach the semi-insulating regime for the target presented. For top –10 polymers, the MCP could generate 10 unique polymers, out of which, although all SMILES could be parsed with RDKit, only 4 had two [*].

**Power Conversion Efficiency (OPV** – The most suitable polymer within 10% tolerance of 4.2; ranging between 3.78–4.62 with SA score of 4.43. For top polymer search with LLM reasoning , polymer with amide linkages (NC(=O)) was generated with OPV of 3.95 (Target: 4.2, Tolerance: ± 0.42 [3.78-4.62]) For top–10 polymers, the MCP could generate 6 unique polymers, out of which, although all SMILES could be parsed with RDKit, only 4 had two [*].

**Dielectric Constant (EPS)** – For one shot polymer search with low SA score, the agent could only reach aliphatic chains for EPS targeted to reach 2.5 even after employing double the average number of tool calls for 1 polymer generation, with high synthetic accessibility score of 4.15. For top polymer search with LLM reasoning, the predicted value of the generated polymer was still near 3.08, where LLM web search suggested a systematic baseline offset in the agentic tools. For top–10 polymers, the MCP could generate 9 unique polymers with 10% tolerance to target value, out of which, although all SMILES could be parsed with RDKit, only 5 had two [*].

This ablation study concludes that the LLM reasoning introduces web-search to modify the SMILES with specific functional groups aiding the polymer search for the agent. To ground the results with the models trained on polymeric databases, the agentic tools only apply modifications in SMILES from the LLM reasoning and uses pretrained models to predict the final property.

# S2. MCP results: prompt design for tool adherence

Examples of prompts in figure S1 show the server generating and using all the tools of the MCP servers. Users can use the same prompt template to generate valid results, as designed by the authors.

**Terminal output**

**USER PROMPT:**

**Design a polymer with an electron affinity of 2 eV, following the steps:**

1. **Generate the initial smiles with the generation MCP**
2. **Validate the polymer property from the prediction MCP**
3. **Use the gemini LLM can change any modificaions , by validationg the resultant property from prediction mcp, try only two modifications**

**MCP Final Response:**

I have completed the design process with the following results:

1. Initial Generation
 * Selected Candidate: *CCCS(=O)CCC(=O)OC(=NC(=CN*)C(=O)O)OC=CF
 * Target Eea: 2.0 eV

2. Validation (Prediction)
 * Predicted Eea: ~2.71 eV
 * Status: The value is higher than the target (2.0 eV).

3. Modifications & Validation
I attempted two structural modifications to lower the Electron Affinity (make the molecule less electrophilic):

Figure S1: Use-case of Polymer-Agent with final MCP response after a sample user query. Note that the user receives a prompt to move forward from the MCP server before any tool is executed.

**Terminal output**

- Modification 1: Removed the Fluorine (F) and the sulfoxide oxygen (S(=O) -> S).
- SMILES: *CCCSCCC(=O)OC(=NC(=CN*)C(=O)O)OC=C
- Predicted Eea: ~2.67 eV (Slight improvement)
- Modification 2: Further removed carbonyls (C=O) and the ester group.
  SMILES: *CCCSCCCC(=NC(=CN*))OC=C
  Predicted Eea: ~2.89 eV (Regressed - Eea increased)

Conclusion:
The initial candidate had an Eea of 2.71 eV. The first modification slightly lowered it to 2.67 eV, but the second modification unexpectedly increased it.
Achieving exactly 2.0 eV would require further, more targeted exploration of the chemical space, likely adding stronger electron-donating groups or breaking the conjugated system more effectively.

Figure S2: Use-case of Polymer-Agent with final MCP response after a sample user query. Continued terminal output from figure S1

Prompts in figure S3 are for generating and using all the tools for targeting single property optimization in Polymer-Agent.

For further validation of the agent's output, we tested the prompts using the specified tools (figure S4, S5) to generate SMILES and validate the output. The same SMILES is then compared with predictions from articles using open-source models, molecular dynamics, and other techniques in the results section.

### Terminal output

**USER PROMPT:**

**Design a polymer targeting an Electronic Conductivity of -4 (log S/cm) and Electron Affinity of 3 eV.**
**Following the steps:**

1. **Generate the initial smiles with the generation MCP**
2. **Validate the polymer property from the prediction MCP**
3. **Use the Gemini LLM can change any modificaions, by validating the resultant property from the prediction MCP, try only two modifications**

**MCP Final Response:**

After optimizing and exploring conjugated scaffolds (Polythiophene, Thiazoles, NDI), I found that these two properties often trade off. High conductivity (PE_I ~ -4) is found in simple donors like Polythiophene but comes with low Eea (~1.3 eV). High Eea (>3 eV) is found in non-conjugated or highly electron-deficient scaffolds but often results in lower conductivity (PE_I < -5.5).

The best balanced candidate is a Fluorinated Polynaphthalene Diimide (PNDI-F) derivative, which achieves the Eea target while maintaining the best possible conductivity for that affinity level.

Best Candidate: Fluorinated PNDI Derivative

- SMILES: *N1C(=O)c2c(F)c3c(c(F)c2C1=O)C(=O)N(C)C3=O*

### Terminal output

- Predicted Properties:
  Electron Affinity (Eea): 2.91 eV (Very close to target 3.0 eV)
  Conductivity (PE_I): -4.97 (Target -4.0; closer than other high-affinity candidates)

Design Process & Trade-offs:

1. Initial Optimization: Found scaffolds with Eea ~3.3 eV but very low conductivity (PE_I ~ -5.8).
2. Conjugated Scaffolds: Tested Polythiophene (*c1ccsc1*), which hit the conductivity target (PE_I = -4.17) but failed the affinity target (Eea = 1.32 eV).
3. Rational Design: I used Naphthalene Diimide (NDI) as a strong electron-acceptor core to boost Eea and added Fluorine atoms (F) to further fine-tune it. This successfully raised Eea to ~2.91 eV while maintaining a higher conductivity than the initial candidates.

The results have been saved to optimized_polymers/final_design.json.

Figure S3: Representation of the Terminal output for a multi-property target given to the MCP server, the results can be saved in any form as per the user in order to reuse the information obtained from the MCP server.

**Terminal output**

**USER PROMPT:**

**Design a polymer targeting an bandgap of 3 and Electron Affinity of 3 eV.**
**Following the steps:**

1. **Generate the initial smiles with the generation MCP**
2. **Validate the polymer property from the prediction MCP**
3. **Report SA score to the user**

**MCP Final Response:**

So my workflow is :

1. Generate using optimize_polymer_properties (OMG model).
2. Validate using predict_all_properties (TransPolymer model).
3. Calculate SA score using sa_score_smiles.

I will now proceed with generating the polymers. I'll use the constraints Eea=[2.9, 3.1] to target the requested 3 eV .

**Terminal output**

I will select the top candidates that have a valid structure. The promising ones are:

1. *NC=NNN=CC(Cl)CSCCOC(Cl)(Cl)OC1CCCC12CCOC(C)(C)O2
2. *C(=O)CC=C(Br)c1ccccc1C=NOC(=O)OCC=C1SS(=O)(=O)C1*
3. *CN1C(=O)NC2C=CC=C21

Now I will:

1. Calculate SA scores for these 3.
2. Validate their properties using predict_all_properties.

The second polymer, *C(=O)CC=C(Br)c1ccccc1C=NOC(=O)OCC=C1SS(=O)(=O)C1*, is a very strong candidate!

* Target Egb: 3.0 eV | OMG: ~2.8-3.0 (implied) | TransPolymer: 2.84 eV
* Target Eea: 3.0 eV | OMG: 3.09 eV | TransPolymer: 2.94 eV
* SA Score: 5.01 (Moderate complexity)

The other two failed validation significantly (Egb > 5 eV, Eea < 1 eV).

I will construct the final report with these details.

Figure S4: Representation of the Terminal output for a multi–property target given to the MCP server, the results are appended with SA score for users.

**Terminal output**

```
Polymer Generation and Validation Report
========================================
Target Properties:
- Bandgap (Egb): 3.0 eV
- Electron Affinity (Eea): 3.0 eV

Top Candidate:
SMILES: *C(=O)CC=C(Br)c1ccccc1C=NOC(=O)OCC=C1SS(=O)(=O)C1*
Assessment:
This polymer shows excellent agreement between the generative model's target and the independent validation model
(TransPolymer).

1. Synthetic Accessibility (SA) Score: 5.01
(Scale: 1 = easy to synthesize, 10 = very difficult. 5.01 indicates moderate synthetic complexity.)
2. Validated Properties (TransPolymer Predictor):
   - Bandgap (Egb):          2.84 eV   (Target: 3.0 eV, Error: 0.16 eV)
   - Electron Affinity (Eea):  2.94 eV   (Target: 3.0 eV, Error: 0.06 eV)
   - Ionization Potential (PE_I): -5.26 eV
   - Dielectric Constant (EPS): 4.70
   - OPV Efficiency (PCE):     3.76 %
```

Figure S5: Representation of the Terminal output for the final property report.

The results mention a user search for a dielectric constant with 3–3.5. The below terminal output, figure S6 shows the final one shot reply and related structures drawn with rdkit library, as image generation is not a part of Polymer-Agent yet.

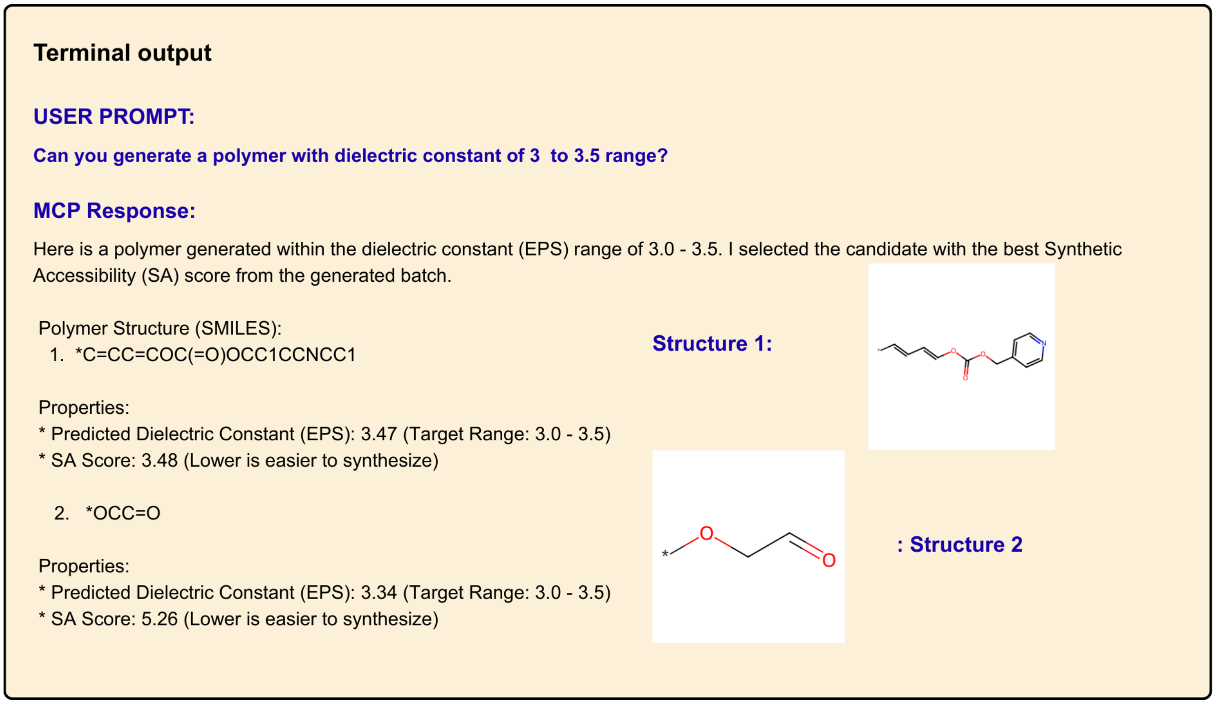


Figure S6: Representation of the Terminal output for the final property report for a dielectric constant range of 3–3.5. Note that second polymer is parsed by RDKit but still contains only one asterisk symbol, and hence is not a valid polymer smiles.

# References

(1) Qiu, H.; Sun, Z.-Y. On-demand reverse design of polymers with PolyTAO. *npj Computational Materials* **2024**, *10*, 273, Publisher: Nature Publishing Group.
